# Supplementary material for: Two-dimensional charge order stabilized in clean polytype heterostructures
Source: Nat Commun. 2022 Jan 20;13:413. doi: 10.1038/s41467-021-27947-5 (PMC8776735; doi:10.1038/s41467-021-27947-5)
Supplement: Supplementary file 1 — Supplementary Information [file 41467_2021_27947_MOESM1_ESM.pdf]

Supplementary Information for

Two-dimensional charge order stabilized in clean polytype heterostructures

Suk Hyun Sung, Noah Schnitzer, Steve Novakov, Ismail El Baggari, Xiangpeng Luo, Jiseok Gim, Nguyen M. Vu, Zidong Li, Todd H. Brintlinger, Yu Liu, Wenjian Lu, Yuping Sun, Parag B. Deotare, Kai Sun, Liuyan Zhao, Lena F. Kourkoutis, John T. Heron, and Robert Hovden

S1 Schematic Diagram of tC-CDW in Interleaved Polytypic Heterostructure

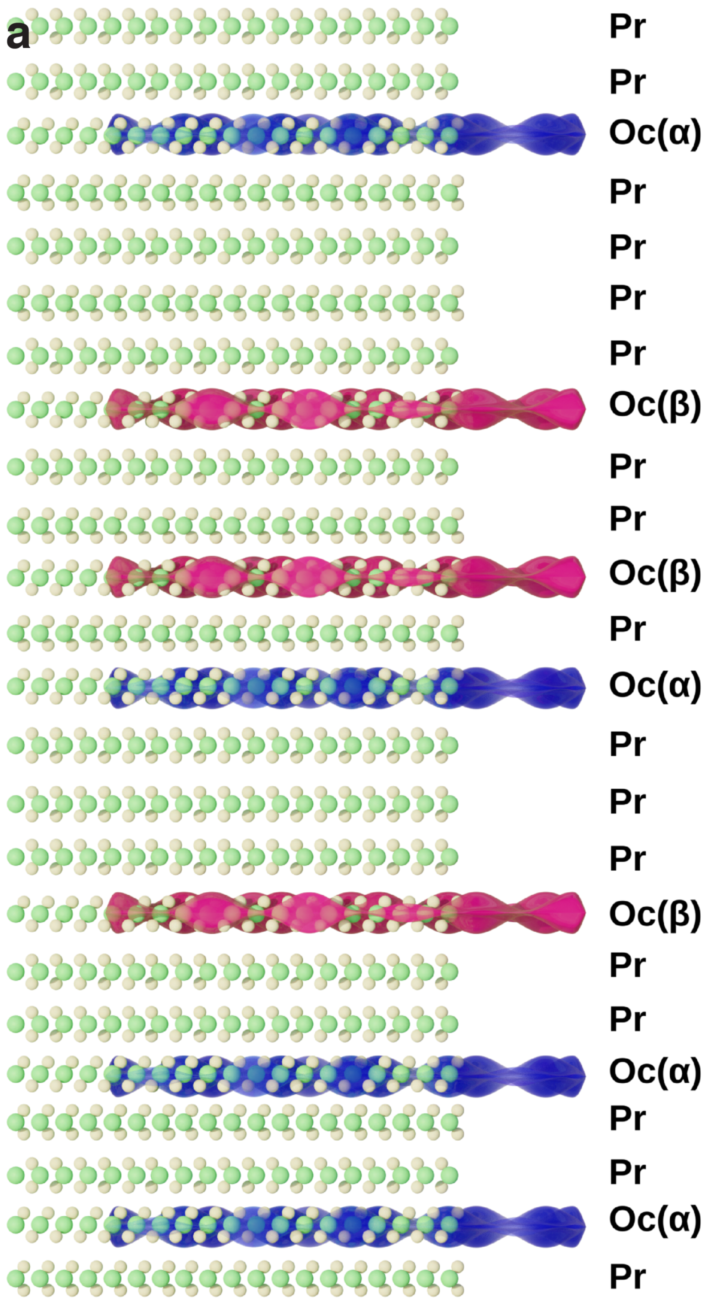

Fig. S1 | Schematic Diagram of tC-CDW in Interleaved Polytypic Heterostructure a) Octahedral layers are sparsely interleaved in between metallic prismatic layers and hosts a C-CDW. Colored overlay represents α (blue) and β (magenta) C-CDW hosted in each octahedral layer. This schematic represents one possible configuration where α and β CDW orientation in each layer occurs with a random, equal likelihood.

S2 Polytypes of TaS_2

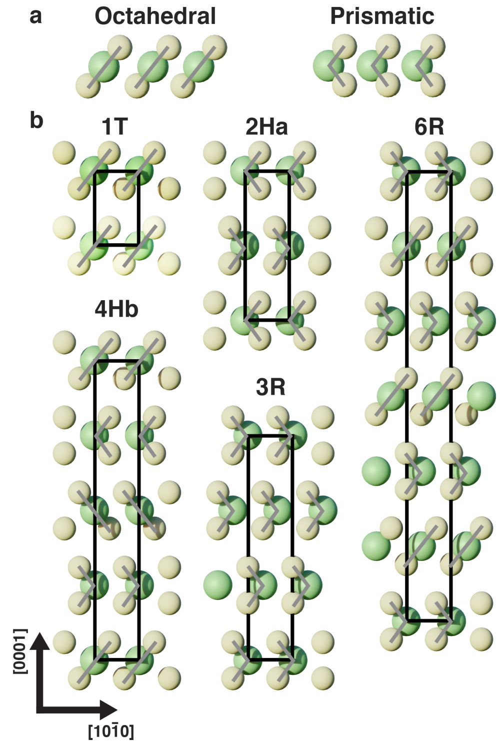

Fig. S2 | Unit cells of previously synthesized TaS_2 polytypes a) Intralayer Ta–S coordination for octahedral (left) and prismatic (right) coordination. Gray lines indicates Ta–S bonds. b) Unit cells of TaS_2 polytypes 1T, 2Ha, 3R, 4Hb and 6R along $\langle 11\bar{2}0 \rangle$ [1–4]. Oc layers comprise 1T in bulk, Pr layers comprise 2Ha and 3R, and 4Hb and 6R contain alternating Oc–Pr stacking.

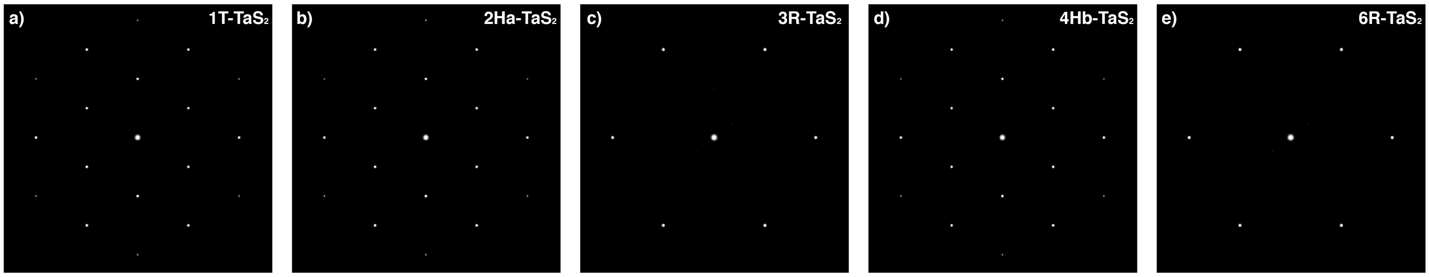

Fig. S3 | Diffraction of TaS_2 Polytypes a–e) Quantum mechanical multislice simulations of SAED for a) 1T, b) 2Ha, c) 3R, d) 4Hb and e) 6R TaS_2 without CDWs. Polytypes and stacking order thereof manifests as distribution of intensities at Bragg peaks. For example, first order Bragg peaks are forbidden reflections for 3R and 6R polytypes due to out-of-plane arrangement. Each simulation was computed for 12 van der Waals layers, with 30 frozen phonon configurations.

S3 Diffraction of PLDs in CDW Materials

Charge density waves (CDWs) are accompanied by periodic lattice distortion (PLD) that displace nuclei positions and manifest as an array of superlattice satellite peaks in diffraction patterns. The reciprocal superlattice structure was first described for single PLD by Overhauser [5] and for any number of harmonics by Hovden et alia [6]. In PLD model, nuclei position ($\mathbf{r}_i'$) from their original positions ($\mathbf{r}_i$) with sinusoidal displacement fields and their n^{th} order harmonics: $\mathbf{r}_i' = \mathbf{r}_i + \sum_n \mathbf{A}_n \sin(\mathbf{q}_n \cdot \mathbf{r} + \phi_n)$. $\mathbf{A}$, $\mathbf{q}$, ϕ denotes displacement vector, wave vector and phase of the modulation wave. Resulted diffraction pattern $\mathrm{I}(\mathbf{k})$ is proportional to squared modulus of the planes waves from each atom:

$$
\begin{aligned}
\mathrm{I}(\mathbf{k}) &\propto \Big| \sum_i \exp[(i\mathbf{k} \cdot \mathbf{r}_i')] \Big|^2 \\
&= \Big| \sum_{i,j} \sum_{\alpha_n} \delta(\mathbf{k} - \mathbf{b}_{i,j} - \sum_n \alpha_n \mathbf{q}_n) \times \prod_n \exp[(i\alpha_n \phi_n) J_{\alpha_n}(\mathbf{k} \cdot \mathbf{A}_n)] \Big|^2,
\end{aligned}
\tag{1}
$$

where $\mathbf{b}_{i,j}$ is reciprocal lattice vector and J denotes Bessel function of first kind.

The δ-function term maps out Bragg peaks (at $\mathbf{b}_{i,j}$) and satellite peaks ($\alpha_n \mathbf{q}_n$) away from Bragg peaks. The second term determines intensity of each diffraction peak. For a typical value of $\mathbf{A}$, $J_{\alpha_n}(\mathbf{k} \cdot \mathbf{A}_n)$ monotonically increases with $\mathbf{k}$. Therefore, CDW superlattice peaks become brighter relative to Bragg peaks near higher order Bragg peaks. In contrast, chemically ordered superlattice peaks retain constant superlattice to Bragg peak relative intensity. Transversity (i.e. angle between $\mathbf{q}$ and $\mathbf{A}$) contributes significantly to the structure of diffraction pattern through $\mathbf{k} \cdot \mathbf{A}$ term.

S4 Superlattice Structure of TaS_2

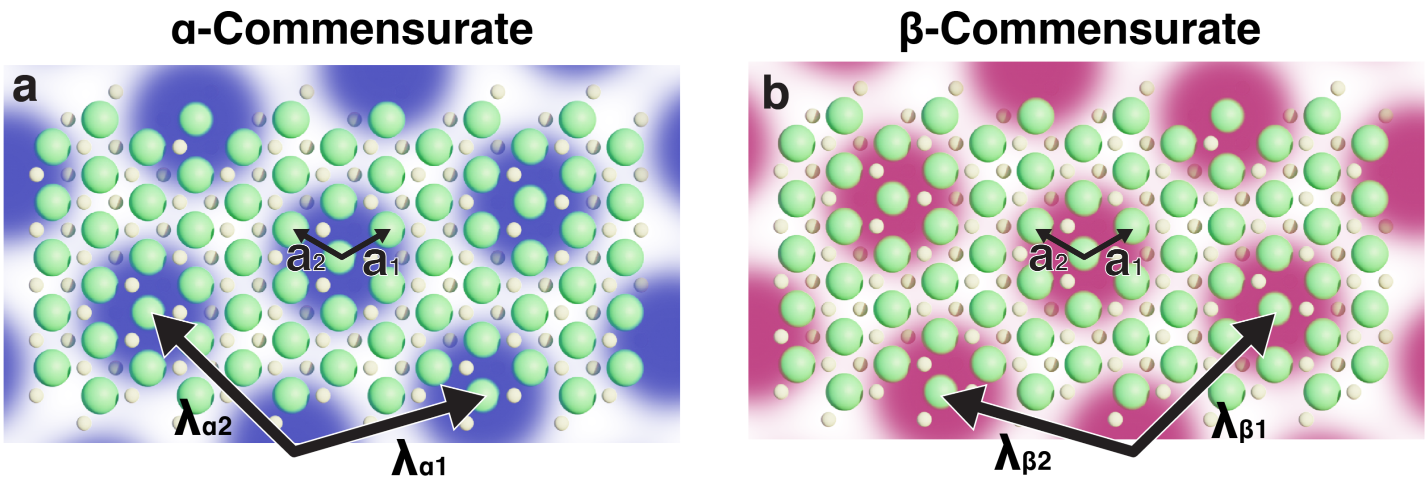

Fig. S4 | Superlattice structure of commensurate CDW in TaS_2 a–b) Schematic diagram describing crystal and superlattice structure of octahedrally coordinated TaS_2 and its associated C-CDW. Large green sphere and small yellow sphere denotes tantalum and sulfur respectively. $\mathbf{a}_1$ and $\mathbf{a}_2$ are lattice vectors defining TaS_2 crystal lattice. α and β C-CDW superlattice is defined by ($\boldsymbol{\lambda}_{\alpha 1} = 3\mathbf{a}_1 - \mathbf{a}_2, \boldsymbol{\lambda}_{\alpha 2} = -4\mathbf{a}_1 + \mathbf{a}_2$) and ($\boldsymbol{\lambda}_{\beta 1} = 4\mathbf{a}_1 - \mathbf{a}_2, \boldsymbol{\lambda}_{\beta 2} = -3\mathbf{a}_1 + \mathbf{a}_2$). Blue and red overlay denotes α and β C-CDW.

S5 In-situ TEM SAED Patterns (Raw data for Fig. 2a)

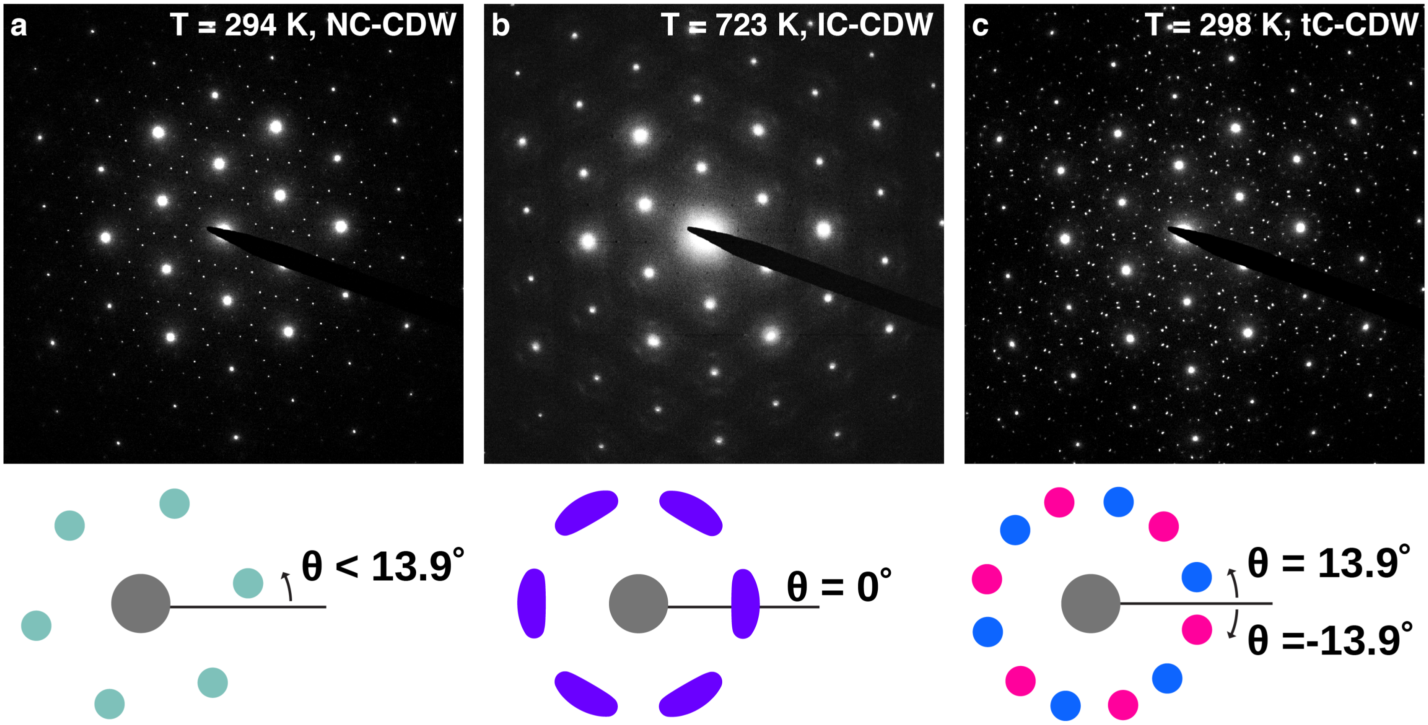

Fig. S5 | In-situ SAED of NC-CDW to tC-CDW evolution. a) NC-CDW phase 1T-TaS_2 SAED pattern features characteristic bright second order and diffused 1st order superlattice reflections. b) At above 350 K, IC-CDW emerges where superlattice peaks are diffused azimuthaly and second order peaks vanishes. c) Heating above 720 K produces interleaved polytypic heterostructure, which hosts a long-range ordered, coherent tC-CDW phase at room temperature

S6 In-situ TEM of TaS_2 polytype transformation

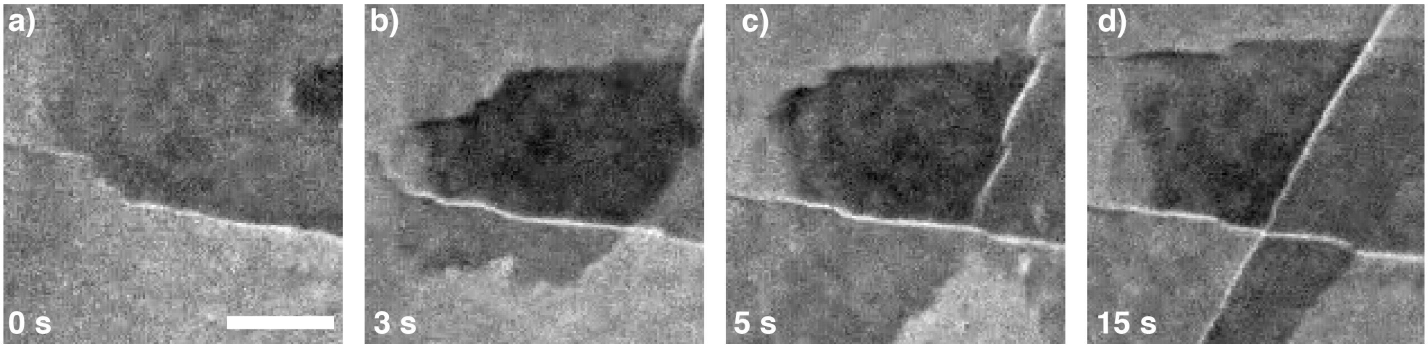

Fig. S6 | In-situ TEM images of layer-by-layer Octahedral to Prismatic polytypic transition In-situ TEM still frames reveal Oc to Pr interpolytypic transition. Domain boundaries propagate without interacting with each other—telltale sign of layer-by-layer transition. The high framerate recording is provided as a Supplementary Movie 1. Scale bar is 350 nm.

S7 Cross-sectional HAADF-STEM

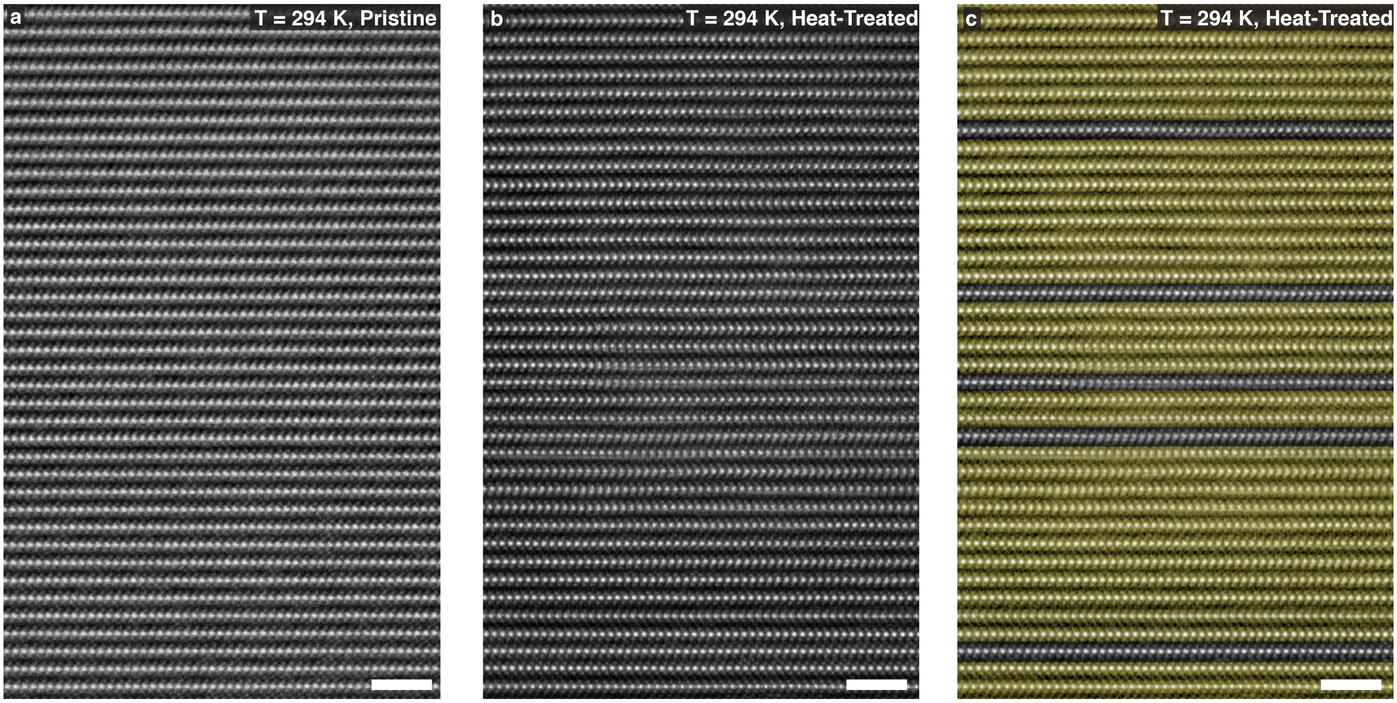

Fig. S7 | Polytypic transition revealed by cross-sectional HAADF-STEM. $[10\bar{1}0]$ cross-sectional HAADF-STEM of TaS_xSe_{2-x} of a) pristine, 1T and b, c) heat-treated polytypic heterostructure. c) Pr-layers are marked yellow. a) Every layer of 1T-TaS_xSe_{2-x} is initially octahedrally coordinated as expected. b, c) Heat-treated sample shows Oc to Pr polytype conversion in some of layers. Monolayers of Oc-TaS_2 are shielded in between slabs of Pr-layers. Scale bar is 2 nm. A selenium doped sample was imaged to enhance chalcogen visibility and determine coordination.

S8 Cross-sectional SAED

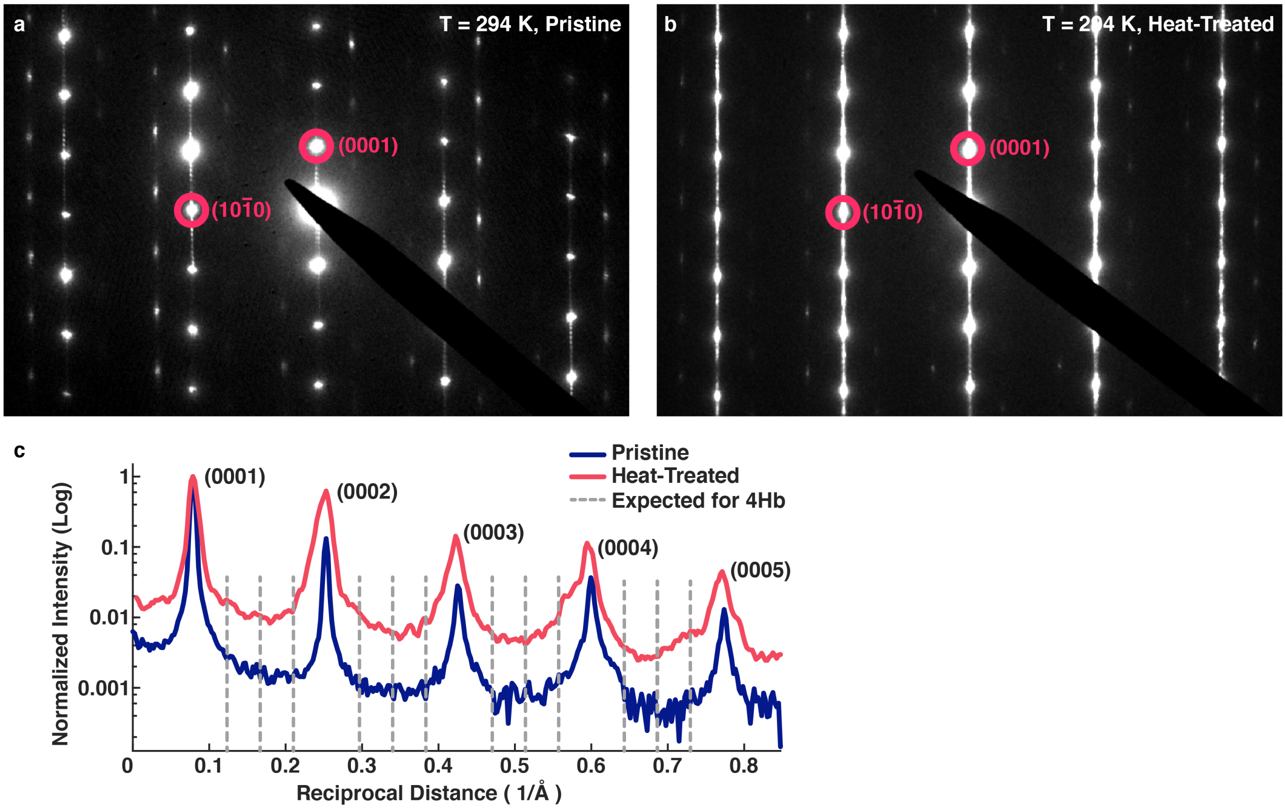

Fig. S8 | Cross-sectional SAED. Cross-sectional SAED pattern of a) pristine TaS_xSe_{2-x} and b) heat-treated polytypic heterostructure. The heterostructure diffraction shows streakings connecting Bragg peaks along [0001], due to disordered polytypic stacking. This is evidently distinct from 4Hb polytype—an ordered bulk crystal phase with alternating Pr–Oc stacking. c) Line profile of pristine (blue) and heat-treated (red) cross-sectional SAED along c-axis. Heat-treated sample shows significant streaking along c-axis. Gray dotted line marks expected Bragg peak location for a 4Hb crystal.

S9 Phenomenological Landau Model for twin degenerate C-CDW

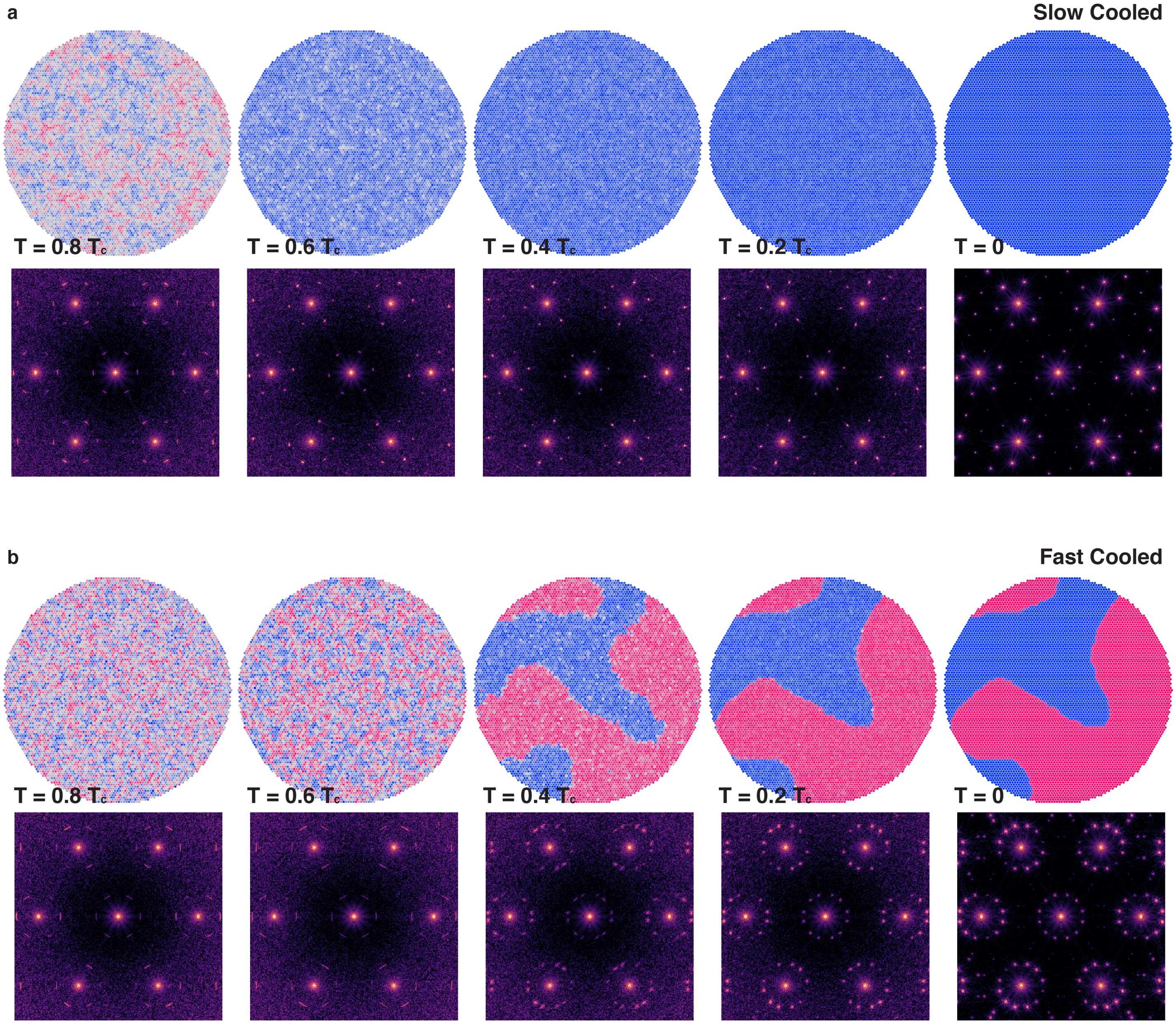

Fig. S9 | Effect of Cooling Rate for Landau CDW. a) $A_2 = 1, A_4 = 10, \gamma = 0.5, 10^{10}$ iterations. b) $A_2 = 1, A_4 = 10, \gamma = 0.1, 10^8$ iterations. In slow cooled simulation (a), all lattice sites converged in to single twin, where as fast cooled simulation (b) shows premature cooling allows for in-plane twin.

S10 NC/C twin CDW

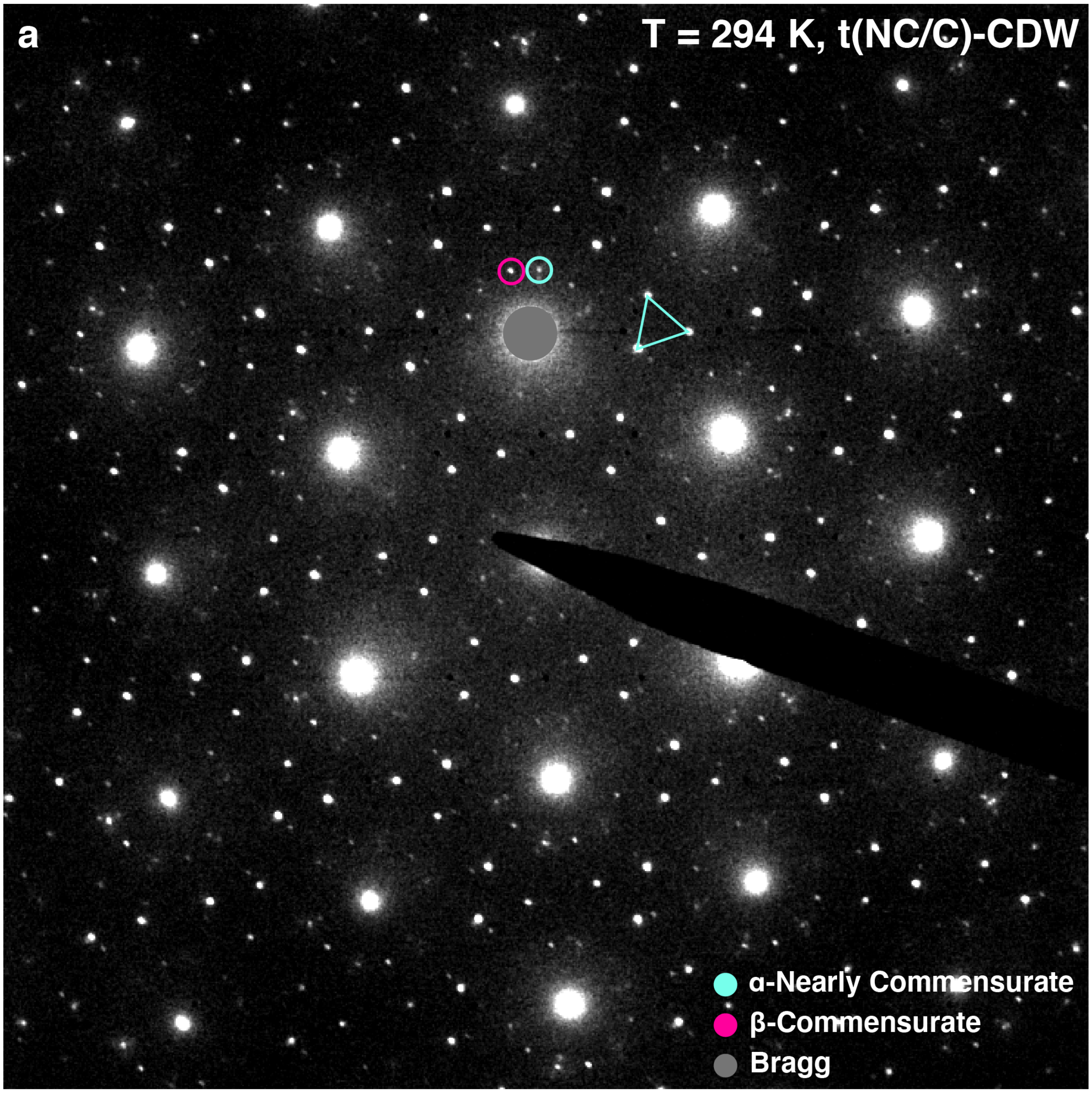

Fig. S10 | NC-C CDW phase in heat-treated TaS_2. a) Room temperature SAED, taken after partial polytypic transitions have occurred, shows both α-NC and β-C CDW peaks where many adjacent (i. e. coupled) CDW layers remain alongside isolated 2D CDW layers, resulting in the presence of both NC and C CDW respectively. Superlattice reflections for NC and C phases are marked cyan and magenta.

S11 Optical Absorption Measurements

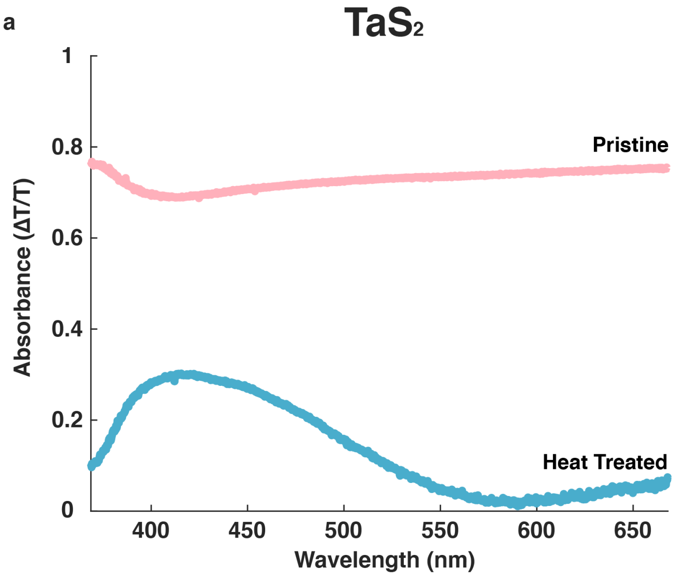

Fig. S11 | Absorption of pristine and thermally treated TaS_2 a) Absorption spectrum at room temperature of pristine NC-CDW 1T-TaS_2 (pink) shows insulating behavior with high absorbance, whereas heat-treated tC-CDW absorption (blue) spectrum shows metallic behavior with low absorption, due to introduction of metallic Pr-layers.

S12 Position Averaged Electron Diffraction Pattern of tC-CDW

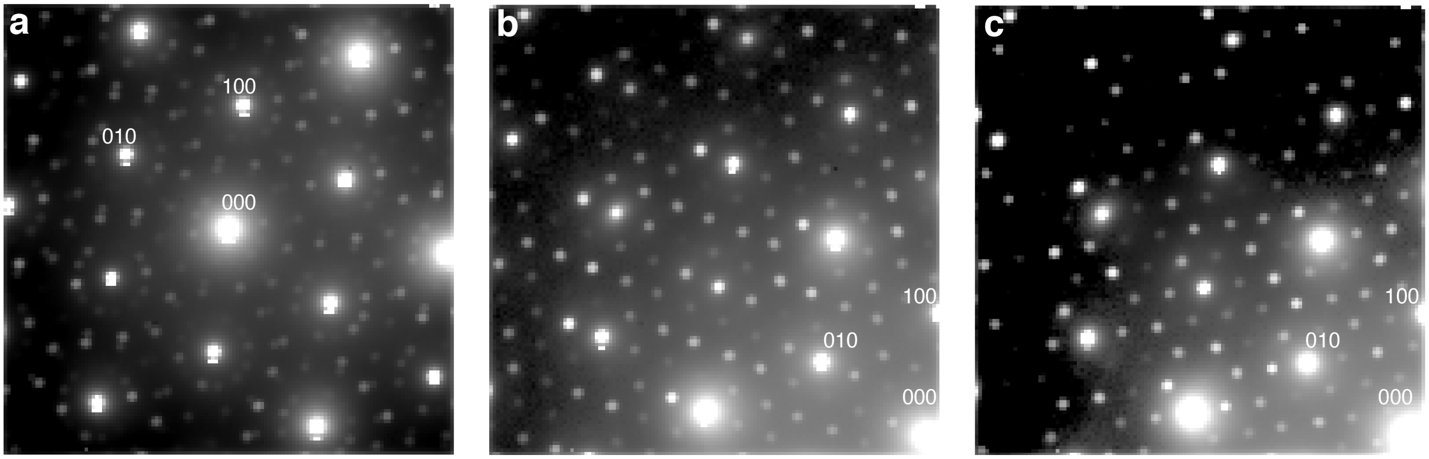

Fig. S12 | Position averaged converged beam electron diffraction (PACBED) pattern of thermally-treated TaS_2 a) Room temperature PACBED pattern of thermally treated TaS_2 reveals tC-CDW superlattice peaks. b, c) Thermally treated TaS_2 in single domain α and β C-CDW, respectively. Because each octahedral layer randomly chooses between α and β upon cooling from IC phase, it is possible for all layers to choose single C-CDW without out-of-plane interaction.

S13 Plan-view Atomic Resolution STEM of 1T and thermally treated TaS_2 and TaSSe

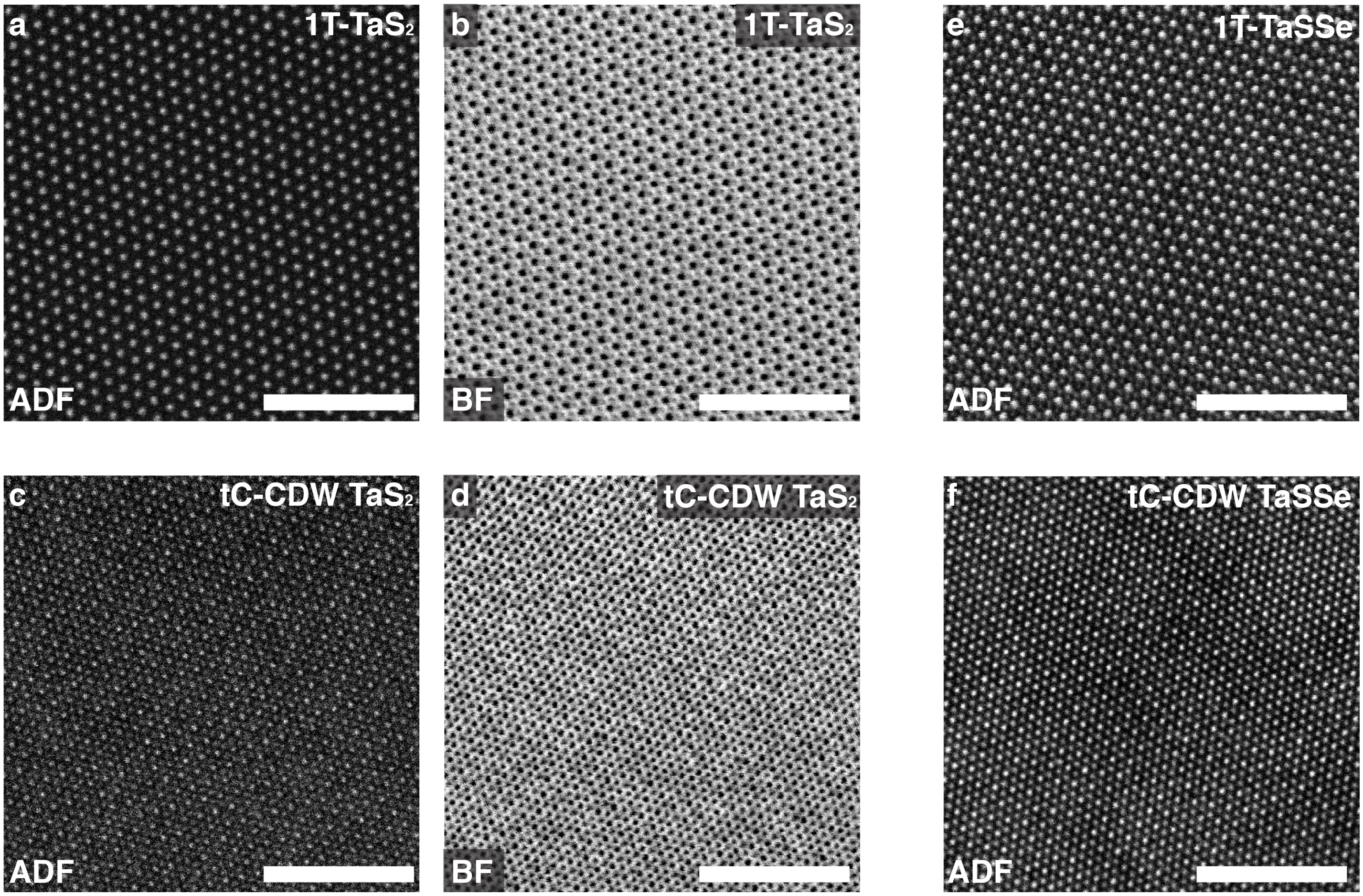

Fig. S13 | Atomic resolution STEM images confirms polytype transformation of TaS_2 and TaSSe a, b) ADF and BF STEM images of pristine 1T-TaS_2. c, d) ADF and BF STEM images of thermally treated tC-CDW TaS_2. Polytype transformation breaks out-of-plane registry. e, f) ADF-STEM of pristine and heat-treated TaSSe, respectively. Polytype transformation occurs independent of Se doping. Scale bars are 3 nm.

S14 SAED of Pristine and Thermally-treated TaSSe

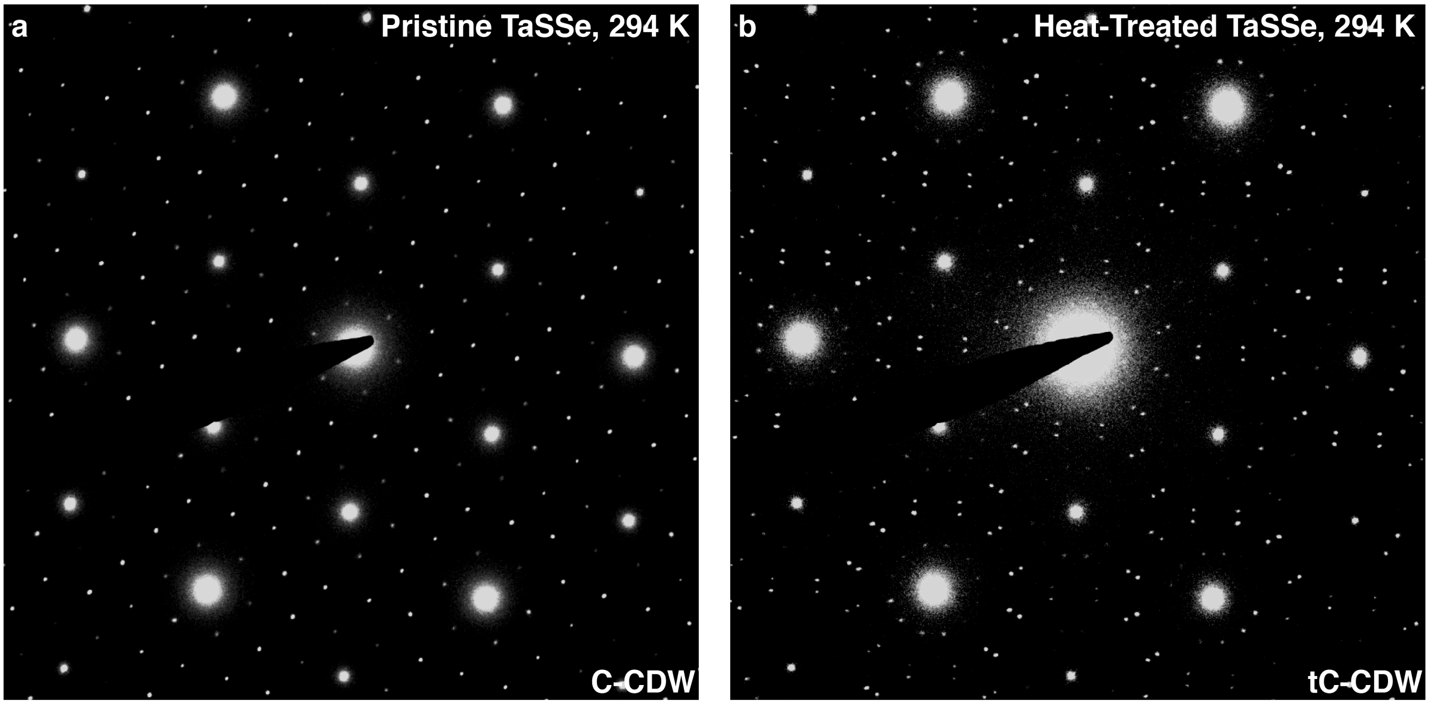

Fig. S14 | Formation of tC-CDW in TaSSea) Pristine 1T-TaSSe shows C-CDW at room temperature. b) Thermally-treated TaSSe shows tC-CDW. Formation of twin occurs independent of Se-doping.

Supplementary References

1. Wilson, J., Di Salvo, F. & Mahajan, S. Charge-density waves and superlattices in the metallic layered transition metal dichalcogenides. *Adv. Phys.* **24,** 117–201 (1975).
2. Jellinek, F. The system of Tantalum-Sulfur. *J. Less Common Met.* **4,** 9–15 (1962).
3. Di Salov, F. J., Bagley, B. G., Voorhoeve, J. M. & Waszczak, J. V. Preparation and properties of a new polytype of tantalum disulfide (4Hb-TaS_2). *J. Phys. Chem. Solids* **34,** 1357–1362 (1973).
4. Thompson, A. H. The synthesis and properties of 6R-TaS_2. *Solid State Commun* **17,** 1115–1117 (1975).
5. Overhauser, A. W. Observability of Charge-Density Waves by Neutron Diffraction. *Phys. Rev. B* **3,** 3173–3182 (1971).
6. Hovden, R. *et al.* Atomic lattice disorder in charge-density-wave phases of exfoliated dichalcogenides (1T-TaS_2). *Proc. Natl. Acad. Sci.* **113,** 11420–11424 (2016).
