# Supplementary material for: Two-dimensional charge order stabilized in clean polytype heterostructures
Source: Nat Commun. 2022 Jan 20;13:413. doi: 10.1038/s41467-021-27947-5 (PMC8776735; doi:10.1038/s41467-021-27947-5)
Supplement: Supplementary file 3 — Description of Additional Supplementary Files [file 41467_2021_27947_MOESM3_ESM.pdf]

File Name: Supplementary Movie 1

Description: **In-situ TEM movie of layer-by-layer Octahedral to Prismatic polytypic transition.** In-situ TEM movie reveals Oc to Pr interpolytypic transition. Domain boundaries propagate without interacting with each other—telltale sign of layer-by-layer transition.
